# Parental Attitudes Toward the Administration of Medicines to Children: A Cross-Sectional Study in Jordan

**DOI:** 10.3390/healthcare14152288

**Published:** 2026-07-28

**Authors:** Abdallah Y. Naser, Hassan Al-Shehri

**Affiliations:** 1 Department of Applied Pharmaceutical Sciences and Clinical Pharmacy, Faculty of Pharmacy, Isra University, Amman 11622, Jordan; abdallah.naser@iu.edu.jo; 2 Department of Pediatrics, College of Medicine, Imam Mohammad Ibn Saud Islamic University (IMSIU), Riyadh 13317, Saudi Arabia

**Keywords:** attitude, children, Jordan, medication, parent, utilization

## Abstract

**Background:** Children are particularly susceptible to the consequences of inappropriate medication use. The practice of parental administration of medicines to children could lead to negative consequences for the child’s health. This study aimed to evaluate parental attitudes in Jordan toward the administration of medicines to children and its associated factors. **Methods:** This is an online, cross-sectional survey study that was conducted in Jordan between November and December 2025 using the Google Forms online tool. The questionnaire tool examined parental attitudes toward the administration of medicines to children. Multiple linear regression was performed to identify demographic factors associated with a more positive parental attitude score toward the administration of medicines to children. **Results:** A total of 1175 parents were involved in this study. Most respondents were female (71.2%) and married (83.5%), with the largest age group being 35–44 years (34.2%). A bachelor’s degree was the most common educational level (40.6%), and nearly half of the participants were employed (43.3%). A total of 55.4% of the parents had a positive attitude toward the administration of medicines to children. The parental attitude scores were significantly associated with female sex, higher income, and having a child aged 3–5 years. Male parents had significantly lower attitude scores toward the administration of medicines to children compared to female participants (B = −2.060, β = −0.075, 95% CI: −3.684 to −0.436, *p* = 0.013). Higher income was significantly associated with more positive attitude scores toward the administration of medicines to children (B = 1.521, β = 0.097, 95% CI: 0.441–2.600, *p* = 0.006). Furthermore, participants with children aged 3–5 years had significantly higher attitude scores toward the administration of medicines to children (B = 1.821, β = 0.066, 95% CI: 0.023–3.618, *p* = 0.047). **Conclusions:** The current study demonstrated a moderate parental attitude toward the administration of medicines to children. Despite positive perceptions regarding the necessity and effectiveness of medications for children, concerns about side effects, drug–drug interactions, and long-term effects were reported.

## 1. Introduction

Because of their distinct physiological development, children are particularly susceptible to inappropriate medication use [1,2]. Such inappropriate medication use in children could result in a variety of health issues, making parental supervision essential when administering children’s prescriptions [3]. Furthermore, the issue of the availability of medications appropriate for children has not yet been resolved, as the proportion of medications tested for pediatric patients is still less than 50% [4]. These factors could increase the risk of medication errors and side effects among children, possibly raising parents’ concerns about the administration of medicines to children.

According to earlier research, the majority of parents support the need for medications but are mostly concerned about their side effects on their children [5,6,7]. For example, in a United States study that was conducted among parents of children with attention deficit hyperactivity disorder (ADHD), 21% of them discontinued the ADHD medication due to psychological side effects and perceived inadequate medication effectiveness [8]. Parents of epileptic patients have also expressed concerns regarding the side effects of antiepileptic drugs, with children’s difficulty concentrating being associated with greater concerns [9]. These concerns could ultimately lead to medication nonadherence, self-medication practices, and a move toward complementary medicines, which might influence children’s long-term behavior and beliefs regarding taking medications because children tend to learn from their parents [10,11]. Additionally, improper medication administration practices could lead to negative consequences for the child’s health, as reported in a prior Saudi study, where 68% of mothers reported self-medicating their children without prior consultation, and 52.7% underestimated their children’s health issues [12].

Despite the availability of previous studies that have looked into parental attitudes and beliefs toward the administration of medicines to children, research that has investigated the factors associated with such beliefs is limited in Arab and Middle Eastern populations. Health beliefs and medicine utilization practices are significantly influenced by cultural, religious, and socioeconomic factors. Consequently, it is possible that findings from Western contexts may not be directly transferable to Arab populations with their unique healthcare systems, cultural norms, and patterns of medicine use. By revealing the factors influencing parental attitudes with regard to the administration of medicines to children, we can provide a baseline for future public health education interventions aiming to improve people’s knowledge and perception of medicine use in children, and emphasize the importance of medication adherence for children’s health and the impact of such practices on their futures. Therefore, this study aimed to evaluate parental attitudes toward the administration of medicines to children and their associated factors in Jordan.

## 2. Methods

### 2.1. Study Design and Setting

This was an online cross-sectional survey study that was conducted in Jordan between November and December 2025 using the Google Forms online tool. This survey utilized the Arabic version of a previously developed questionnaire tool by Hämeen-Anttila et al. [5].

### 2.2. Study Population and Sampling Procedure

Only participants who satisfied the inclusion criteria were included in the study. The study population comprised parents with children below the age of 18. Qualified participants were solicited to engage in this study by the convenience sampling method. The research participants were recruited through social media channels, specifically WhatsApp and Facebook. The questionnaire link and the study invitation letter were disseminated across several social media platforms. The social media groups and sites were chosen for their diversity, incorporating people from various sociodemographic backgrounds. The aim was to connect with parents from a variety of sociodemographic contexts in Jordan, and groups were chosen based on their accessibility and active membership. Recruitment across numerous groups of varying community types was intended to reduce the homogeneity of the sample, despite the absence of formal probability-based group selection. The invitation letter specified the study’s inclusion criteria and requested participation only from individuals who met them.

### 2.3. Study Instrument

The questionnaire tool comprised four parts. The first part recorded the sociodemographic characteristics of the participating parents, including the parents’ sex, age, highest educational level completed, occupation, marital status, and family monthly income (in Jordanian dinar; where 1 JOD equals 1.41 USD). The monthly income categories were defined based on the national average monthly income [13]. The second part recorded their children’s information, including their ages and family size. The third part recorded the families’ children’s health and disease history, including whether the children currently have any chronic illnesses; how often they experienced acute illnesses; whether they were hospitalized in the past 12 months; whether a physician was consulted before they were prescribed medicines; whether the children were ever given over-the-counter (OTC) medicines without consulting a doctor; the families’ main source of information about children’s medicines; and whether the parents worked in a healthcare profession. The fourth part examined parental attitudes toward the administration of medicines to children, utilizing 18 items with a 5-point Likert format scale (I agree completely = 5; I agree = 4; neutral = 3; I disagree = 2; I disagree completely = 1). In addition, a “no opinion = 0” answer was possible, which was adapted from the previously published literature by Hämeen-Anttila et al. [5]. To capture stable and generalized parental attitudes toward the administration of medicines to children, rather than being asked for situation-specific responses associated with a single child, parents were requested to respond to attitude items regarding their administration of medicines to children without specifying a particular referent child, but based on their overall experience.

To prevent systematic downward bias in the total attitude scores, responses that indicated “no opinion” were recoded as 3 (neutral) before all analyses: see the questionnaire tool in the Supplementary Materials. Eleven items reflecting cautious, restrictive, or concern-based attitudes were reverse-coded so that consistently higher overall scores reflected a more positive parental attitude towards the administration of medicines to children. The maximum possible score was 90: the higher the score, the more positive the parental attitude toward the administration of medicines to children, reflecting greater acceptance, trust in medicines, and reduced avoidance behavior (which does not inherently imply clinically optimal medicine utilization behavior). We chose to employ a single total composite attitude score due to the high overall internal consistency of the 18-item scale in our sample (Cronbach’s α = 0.926), in accordance with other studies that have utilized composite attitude scores from multi-domain Likert scales.

The questionnaire was translated from English to Arabic using the forward–backward translation method. The questionnaire was independently translated into Arabic by two bilingual researchers with pharmacy expertise. The two Arabic versions were compared and consolidated into a single reconciled Arabic version. A third independent bilingual translator then back-translated the agreed-upon Arabic draft into English. The resulting English version was compared to the original questionnaire to confirm conceptual equivalence and accuracy. An expert panel subsequently evaluated the Arabic-translated version by evaluating its content validity, instrument clarity, and cultural appropriateness. The panel, consisting of a pediatrician and a clinical pharmacist, endorsed the questionnaire. The content validity of the tool was checked following an evaluation of its language accuracy for the intended study population. A pilot study was subsequently undertaken, using 30 participants from the designated study population; the study similarly validated the clarity of the survey items.

The internal reliability of the scale was evaluated using Cronbach’s alpha, yielding a score of 0.926, indicative of excellent internal consistency. The items of the original questionnaire remained unaltered. To investigate the factor structure of the 18 items of the questionnaire, principal component analysis (PCA) with Oblimin rotation was implemented. The data were appropriate for factor analysis, achieving a significant Bartlett’s test of sphericity (χ^2^(153) = 11,124.68, *p* < 0.001) and a Kaiser–Meyer–Olkin measure of sampling adequacy (KMO = 0.937). Three components with eigenvalues greater than 1 were extracted, which accounted for 61.35% of the total variance. The conceptually meaningful three-factor structure that resulted from the rotated solution was as follows: (1) beliefs and concerns regarding medications (eight items); (2) medication avoidance and naturalistic beliefs (seven items); and (3) confidence in OTC medications (three items). The extracted factors adequately represented all items, as the communalities ranged between 0.506 and 0.734.

The PCA revealed a multidimensional scale structure consisting of three distinct factors. Nevertheless, the rationale for employing a single composite total score was as follows: the scale’s strong general attitude factor was suggested by the overall Cronbach’s α of 0.926, which indicated exceptional internal consistency across all 18 items. Furthermore, given the moderate to moderately strong observed correlation between the three components (component correlation matrix range: r = 0.362–0.505), this confirms that these three factors are related dimensions of a broader underlying construct of parental attitude toward administration rather than independent domains, which further substantiates the use of an overall attitude score and recognizes the multidimensional character of the scale.

### 2.4. Sample Size Calculation

The Raosoft (https://raosoftcalculator.net/) online web-based calculator was used to estimate the minimum required sample size for this study. Based on the statistics of the Department of Statistics in Jordan, there are approximately 2.47 million households (which was used as an approximation of the target population size) in Jordan in 2025 [14]. To estimate the minimum required sample size, we used the following formula:

N = Z^2^p (1 − p)/d^2^, where N is the minimum required sample size; Z is the value for the 95% confidence interval, which was equal to 1.96; the expected proportion is included (which was estimated to be 0.5 to maximize the required sample size and increase the statistical power); and d is the margin of error (which was equal to 0.05). Based on the above, the minimum required sample size was 385 parents.

### 2.5. Patient and Public Involvement

It was not appropriate or possible to involve patients or the public in the design of the study, or how it was administered, reported, or how our research plans were disseminated.

### 2.6. Ethical Approval and Informed Consent

Ethical approval for this study was obtained from the Scientific Research Ethics Committee of Isra University, Amman, Jordan (SREC/25/11/170). This study was conducted in accordance with the World Medical Association Declaration of Helsinki. Informed consent was obtained from the study participants before completing the study questionnaire.

### 2.7. Data Analysis

All analyses were performed using the Statistical Package of the Social Sciences (SPSS version 28). Categorical data, such as the demographics and health-related characteristics, were reported as frequencies and percentages, while mean, median, standard deviation (SD), and range were utilized to summarize the continuous data, such as the attitude scale. The Kolmogorov–Smirnov test was applied to assess the normality of the total attitude score, and the data met the assumptions of parametric analysis. Accordingly, Student’s *t*-test and one-way Analysis of Variance (ANOVA) were conducted when applicable. Multiple linear regression was performed to identify demographic factors associated with parental attitude towards the administration of medicines to children. Results of the multiple linear regression analysis were reported as both unstandardized coefficients (B) and standardized coefficients (β) with corresponding confidence intervals (CIs). The dependent variable in the regression model was the overall parental attitude score. The independent variables in the regression model were sociodemographic and health-related characteristics of the study participants. A *p*-value less than 0.05 was considered statistically significant.

To evaluate multicollinearity among independent variables, tolerance and variance inflation factor (VIF) statistics were employed, which were estimated from the linear regression model. The absence of problematic multicollinearity was confirmed by the fact that all variables exhibited acceptable collinearity, with tolerance values ranging from 0.580 to 0.952 and VIF values ranging from 1.050 to 1.725. Furthermore, the maximum condition index was 12.876, which was also well below the threshold of 30. A complete case analysis was achieved as no missing data were observed across any of the study variables. The Hosmer–Lemeshow goodness-of-fit test was employed to evaluate the model’s fit, and Nagelkerke’s R^2^ statistic and the overall classification accuracy of the model were used to summarize its performance.

## 3. Results

### 3.1. Participant Characteristics

A total of 1175 parents were involved in this study. Most respondents were female (837, 71.2%) and married (981, 83.5%), with the largest age group being 35–44 years (402, 34.2%). A bachelor’s degree was the most common educational level (477, 40.6%), and nearly half of the participants were employed (509, 43.3%). Most households reported a monthly income of between 500 and 1000 JOD (507, 43.1%), as seen in Table 1.

The majority reported no child comorbidities (928, 79.0%). Physicians (566, 48.2%) and pharmacists (446, 38.0%) were primary sources of information on pediatric medications, and most parents in our study sample reported consulting a physician often or always before giving medication to their children (589, 50.1%), see Table 2.

### 3.2. Distribution of Parental Attitudes Toward the Administration of Medicines to Children

**Figure 1:**
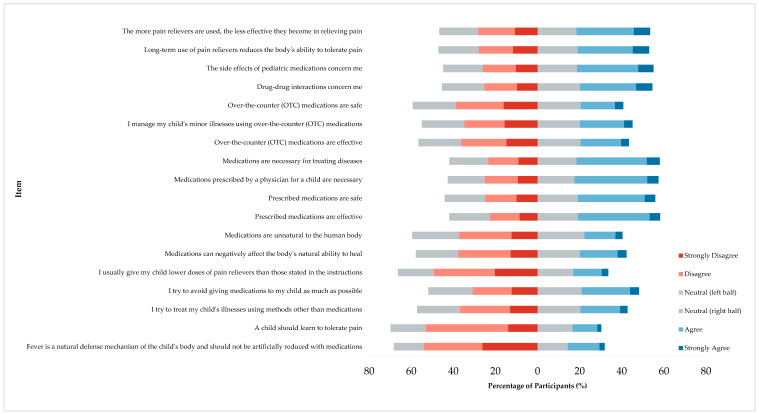
Distribution of parental attitudes toward administration of medicines to children.

Agreement or strong agreement that prescribed medications are effective and necessary was reported by 457 (38.9%) and 468 (39.9%) parents, respectively. Concerns about medication safety were evident, as 427 (36.3%) parents agreed or strongly agreed that drug–drug interactions are concerning and 427 (36.3%) expressed concern about the side effects of pediatric medications. Regarding pain management, 398 (33.9%) parents agreed or strongly agreed that long-term use of pain relievers reduces the body’s ability to tolerate pain, and 411 (35.0%) believed that increased use of pain relievers reduces their effectiveness, see Figure 1.

### 3.3. Comparative Analysis of Parental Attitude Scores

**Figure 2:**
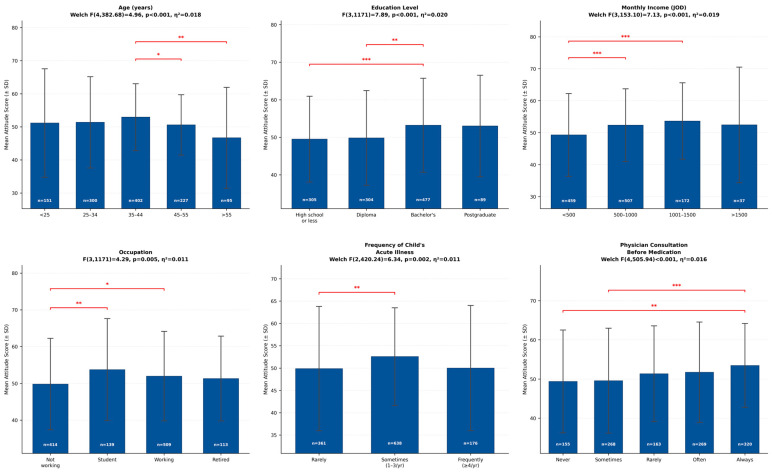
Mean parental attitude scores with post hoc pairwise comparisons. Error bars represent ± 1 SD. * *p* < 0.05, ** *p* < 0.01, *** *p* < 0.001. η^2^ = eta squared (effect size).

Independent t-tests or Welch’s ANOVA with post hoc tests were applied as appropriate to compare parental attitude scores across demographic groups. The reported mean parental attitude score in our study sample was 51.36 ± 12.45 out of 90, reflecting a moderate attitude toward the administration of medicines to children. Female parents had significantly higher attitude scores than males (51.94 ± 11.93 vs. 49.93 ± 13.58, *p* = 0.012). Attitude scores differed significantly by age (*p* < 0.001), with the highest mean observed among parents aged 35–44 years (52.96 ± 10.10) and the lowest mean among those older than 55 years (46.69 ± 15.26). Higher scores were also found among participants with a bachelor’s degree (53.21 ± 12.52) or postgraduate education (53.02 ± 13.52) compared with those with lower education levels (*p* < 0.001), see Figure 2.

### 3.4. Predictors of Parental Attitude Toward the Administration of Medicines to Children

The multiple linear regression model was statistically significant (F(13, 1161) = 4.11, *p* < 0.001), despite accounting for only a modest proportion of the variance in attitude scores (R^2^ = 0.044, adjusted R^2^ = 0.033), showing that the included variables in the regression model explain only 4.4% of the variability in parental attitude, and that the remaining variance could be due to unmeasured variables that are not measured in the present study. The parental attitude scores were significantly associated with female sex, higher income, and having a child aged 3–5 years. However, the standardized regression coefficients for all significant predictors were small in magnitude (β range: −0.075 to 0.097), showing that while these associations are statistically detectable, their practical significance is limited and should be interpreted with caution. Male participants had significantly lower attitude scores toward the administration of medicines to children than female participants (B = −2.060, β = −0.075, 95% CI: −3.684 to −0.436, *p* = 0.013). Higher income was significantly associated with higher attitude scores (B = 1.521, β = 0.097, 95% CI: 0.441–2.600, *p* = 0.006). Furthermore, participants with children aged 3–5 years had significantly higher attitude scores (B = 1.821, β = 0.066, 95% CI: 0.023–3.618, *p* = 0.047). The remaining predictors did not reach the statistically significant level (*p* > 0.05), see Table 3.

## 4. Discussion

The purpose of the current study was to evaluate Jordanian parents’ attitudes toward the administration of medicines to children and investigate the factors that were associated with those attitudes. The study’s findings will provide crucial insights for future public awareness campaigns aimed at improving people’s perceptions of medicines and the importance of medication adherence for themselves and their children.

It must be acknowledged that, in the current study, parents’ preference for physicians (48.2%) and pharmacists (38.0%) as primary sources of medicine information is indicative of their general confidence in professional guidance and should be interpreted as conceptually distinct from episode-specific consultation behavior. For example, 27.1% of our study participants reported that they rarely or never consult a physician before giving medication to their child. The finding that a portion of parents reported administering OTC medications without consulting a physician does not contradict their stated preference for professional information sources. Rather, it reflects the pragmatic reality that parents frequently self-manage minor childhood conditions without seeking professional consultation for each unique episode.

The current study found that, in our study sample, fewer than half of the participants perceived the prescribed medications for children as effective (38.9%) and necessary (39.9%). In comparison, more than three-quarters of the parents in another Jordanian study believed that prescribed medications were effective (78%) and necessary (82%) for their children [6]. Similarly, around 84% of Finnish parents reported feeling that prescribed medications were effective for children, whereas only half believed the same about over-the-counter medications, likely due to their trust in doctors who prescribe them [5]. In the US, 72% of parents reported believing that their children’s asthma treatments were necessary; however, 30% of them expressed serious worries about the medications [15]. Similar patterns of concern were reported in our study: 36.3% of parents expressed concerns about drug–drug interactions and the side effects of pediatric medications. In a prior study involving 706 parents of children using daily asthma medications, 38.2% of them expressed concerns about these medications and their side effects, such as growth retardation (48.9%) [16]. According to a different survey of 1000 parents in Jordan, 80% of them were worried about the adverse effects and interactions between medications, and nearly 60% tried to avoid giving their children medications due to these worries [6]. This underlines the importance of addressing parents’ concerns about medications, especially their side effects, and raising their awareness in terms of medication adherence. Differences in study design, settings, and participants could justify the variability in the proportion of parents who believe in the effectiveness and necessity of prescribed pediatric medications and those who express concerns regarding their use.

In the current study, around one-third of parents believed that frequent long-term use of pain relievers reduces the body’s ability to tolerate pain (33.9%) and reduces their effectiveness (35%). Similarly, a previous study investigating parents’ beliefs about postoperative pain management in children reported that 64% believed that analgesics should be used only when necessary, such as in cases of extreme pain, indicating that using analgesics with caution is a cross-cultural phenomenon that is not exclusive to any particular region or ethnic group [17]. This suggests that parental hesitancy to take analgesics may reflect widely held health beliefs rather than culturally specific attitudes. According to another survey conducted to assess the effect of ethnicity and language on parental attitudes toward using analgesics to manage pain in children, most English-speaking white and Hispanic parents disagreed with the perception that pain medications work the same regardless of the frequency of use [18]. Moreover, nearly half of the parents enrolled in a Finnish study were concerned about the long-term effects of pain relief medications (46%) [5]. These findings illuminate the importance of correcting parental misconceptions and alleviating their concerns regarding pain medications to avoid undertreatment of pain and support optimal pain management in children.

Nearly half of participants in the present study were classified as having a more positive attitude toward the administration of medicines to children (55.4%) based on the overall attitude score (scored above the mean score of the study sample), showing a general orientation toward medicine acceptance rather than uniformly favorable perceptions, with those holding a bachelor’s degree having a significantly greater acceptance compared to those with high school education or less. A study conducted in Finland found that parents with a lower level of education exhibited less favorable attitudes toward medicine use in children and greater concerns regarding their risks [5]. Parents with higher education levels may be more knowledgeable about medications and more aware of their importance for their children’s health, as suggested by previous research. On the other hand, parents with a lower level of education might be less knowledgeable and have misconceptions and concerns about the risks associated with medicine use in children, highlighting the importance of education programs in improving parental views on this [19,20]. However, these explanations present theoretically plausible interpretations, as medication knowledge was not directly assessed in the present study.

In the current study, parental attitude scores toward the administration of medicines to children were substantially associated with female sex, higher income, and having a child aged 3–5 years. In the current study, higher income was significantly associated with higher attitude scores toward the administration of medicines to children (B = 1.521, β = 0.097, 95% CI: 0.441–2.600, *p* = 0.006). Furthermore, participants with children aged 3–5 years had significantly higher attitude scores toward the administration of medicines to children compared to others (B = 1.821, β = 0.066, 95% CI: 0.023–3.618, *p* = 0.047). This is consistent with the findings of a previous study by Hämeen-Anttila et al. [5], which identified that household income was a significant predictor associated with parental attitude toward the administration of medicines to children [5]. Higher household income has been associated with greater access to healthcare services and might be associated with better healthcare literacy, which could be associated with a more positive attitude toward the administration of medicines to children in the previous literature. This is confirmed by the findings of previous studies by Tran et al. and Isacson et al. that found that medicine utilization is associated with multiple health and behavioral factors, including education level, income level, medication knowledge, and self-care orientation [21,22]. However, these factors were not measured in the current study, and this interpretation remains a plausible theoretical explanation. A previous study in Jordan examined parental self-efficacy in managing pediatric medications and treatments, and found that higher parental self-efficacy was significantly associated with parents’ socioeconomic characteristics, including income and number of children [23].

The previous literature identified that a greater acceptance toward OTC medicine may be associated with a more positive attitude toward the administration of medicines to children. As OTC medicines are freely available from pharmacies and do not require prescriptions, they may not be considered as dangerous as prescription-only medicines by the parents, and may be associated with a lower possibility of seeking medical consultation [5]. A previous study by Siponen et al. identified that parents’ greater acceptance toward medicines in general was found to predict use of OTC medicines among children [11]. Furthermore, Siponen identified that OTC medicine and complementary and alternative medicine use were both most common among children whose parents had positive thoughts about OTC medicines. Moreover, they identified that the young age (3–11 years) of a child and a child’s poorer health status, as reported by the parents, were significant predictors of an association of parental attitudes with children’s use of OTC medicines [11].

The previous literature identified the number of children and having hospitalization history in the past 12 months as important predictors that might be associated with a more positive attitude toward the administration of medicines to children. A previous Jordanian study reported that parents with one child were less confident in managing their children’s healthcare tasks, including medications, which might reflect an unfavorable attitude toward the administration of medicines to children [23]. The previous literature also reported that parents with one or two children might have less experience in pediatric care, which may be associated with them being more cautious about giving medications to their children and, in turn, might be associated with their views in this case. The same scenario can occur with parents who have hospitalized children, as fear and anxiety may have developed in these parents associated with this experience, affecting their perception of medical care and leading them to be more cautious and have a lower acceptance toward children’s healthcare—including medications—which has been reported in the previous literature [24].

In our study sample, male parents were less likely to report a more positive attitude toward the administration of medicines to children (B = −2.060, β = −0.075, 95% CI: −3.684 to −0.436, *p* = 0.013). A previous study in Malaysia identified that mothers had a significantly more positive attitude than fathers (*p* < 0.05) [25]. This could be because mothers usually have a common role in taking care of the family’s health, which has been reported in the previous literature [5,25]. Furthermore, our research identified mothers as the main caregivers of their children and that they have better capabilities and skills for the management of their children’s illnesses [6,26]. The statistical significance observed in this study should not be confused with practical relevance. Due to the large sample size of 1175 participants, even small and practically negligible associations may achieve statistical significance, as large samples offer substantial statistical power to detect even small effects. The multiple linear regression model, although statistically significant, accounted for only 4.4% of the total variance in parental attitude, suggesting that the variables examined in this study are weak predictors of parental attitudes. The standardized regression coefficients for all significant predictors were of a small magnitude, which emphasizes the limited practical significance of these associations. Given the substantial inexplicable variance, it is reasonable to assume that parental attitudes are influenced by factors that are not included in the current study.

It should be noted that, in this research, a more positive attitude toward the administration of medicines to children was defined as a single composite attitude score that exceeded the mean attitude score of the study sample. The present study’s results must be interpreted in the context of the multidimensional nature of parental attitudes toward the administration of medicines to children. Item-level data indicated that 38.9% of the parents agreed with statements regarding the necessity and effectiveness of prescribed medications. This indicates that a significant number of parents remain skeptical regarding the fundamental value of medicines, despite them being classified as having a positive overall attitude. In this study population, approximately 36.3% of the parents expressed concern about drug–drug interactions and the side effects of pediatric medications, which is indicative of medicine-related anxiety that can coexist with overall medicine acceptance.

In terms of avoidance and restrictive medication behavior, a significant number of parents expressed support for items that reflect medicine avoidance, such as treating their child’s illnesses using methods other than medications, avoiding the administration of medications to the child as much as possible, and administering pain relievers at lower doses than prescribed. This suggests that restrictive medication behaviors are still prevalent among parents with an overall greater acceptance toward the administration of medicines to children. These item-level patterns collectively underscore the fact that the single composite attitude score, while offering a valuable summary measure, inadvertently conceals significant variability across specific parental attitudinal dimensions. Future research is encouraged to conduct a separate analysis of these dimensions at the subscale level to offer more specific and clinically actionable insights into parental attitudes toward the administration of medicines to children.

### Limitations

The online cross-sectional survey design restricted the ability to explore causality between the study variables. In addition, the implementation of the convenience sampling technique limited the generalizability of the study’s conclusions as this approach introduces selection bias. Parents without internet access, lower digital literacy, or limited engagement with social media are likely to be underrepresented. The sample was also predominantly female, which may affect the generalizability to fathers. Furthermore, we observed overrepresentation of parents working in the medical field in our study sample. However, the regression analysis demonstrated that parental attitude scores were not significantly associated with employment in the healthcare sector, indicating that this overrepresentation did not significantly impact the primary findings. Despite the fact that parents with formal medical training may be expected to exhibit distinct attitudes toward medicine administration in comparison to others, the non-significant observed findings in this research may be attributed to a variety of factors. For example, healthcare professionals may respond to their own children’s health needs primarily through an emotional and protective parental perspective when acting as parents, rather than strictly applying their professional training knowledge. Additionally, parental attitudes toward the administration of medicines to children may be more significantly influenced by cultural beliefs and direct family experience than by formal medical knowledge alone. Equally, the ”healthcare profession” variable in this study included a diverse array of healthcare professionals, including nurses, physicians, pharmacists, and allied healthcare workers, each with a unique level of pharmacological training. Furthermore, non-healthcare parents may possess substantial practical knowledge about common pediatric medicines, thereby reducing the knowledge gap between the two groups.

In addition, self-administered surveys are prone to a social desirability bias and a reporting bias, as the participants might not reflect on their actual attitudes. Due to the fact that the questionnaire link was disseminated across numerous social media platforms in order to facilitate access to a diverse parent population, it was impossible to ascertain the total number of individuals who were exposed to the survey link. This prevented the estimation of a conventional response rate. Rather than a deficiency in data collection procedures, this is a common methodological feature of convenience sampling through open social media dissemination, and it precludes the formal assessment of potential non-response bias. It is important to acknowledge that the utilization of mean score-based classification is sample-dependent and does not represent a clinically validated threshold. Consequently, it is advisable to carefully compare the proportion of individuals classified as having better attitudes across other studies, as the mean cut-off values will vary among samples with varying sociodemographics from different studies. Furthermore, our PCA revealed a three-factor structure, suggesting that the scale is multidimensional despite the fact that the primary analysis in the present study utilized a composite total attitude score, which was supported by the scale’s high overall internal consistency. Consequently, the utilization of a composite score is a deliberate analytical decision rather than an inherent limitation of the instrument. We recognize that subscale-level analysis may offer a more clinically meaningful level of detail regarding specific attitudinal dimensions than a composite total attitude score. Consequently, we suggest that future studies investigate these subscales individually. Furthermore, cognitive interviewing was not performed, and the content validity index was not examined. Moreover, the high Cronbach’s alpha may suggest item redundancy. Consequently, the findings of this research should be interpreted carefully. Future longitudinal studies are needed to examine parental attitudes toward different classes of medications and the influence of the nature of the disease on parents’ behavior.

## 5. Conclusions

The current study demonstrated a moderate parental attitude toward the administration of medicines to children, with more than half of the parents in our sample showing a more positive attitude: higher scores reflected a more positive overall parental attitude toward the administration of medicines to children. Inherently, this classification does not entail clinically optimal or universally safe medication behavior; rather, it is a relative measure of parental attitude toward the administration of medicines to children within this study population. Despite positive perceptions regarding the necessity and effectiveness of medications for children, concerns about side effects, drug–drug interactions, and long-term effects were reported, indicating that medication administration attitudes coexist with meaningful safety-related concerns. Several factors were significantly associated with parental attitude toward the administration of medicines to children in our study sample, including parents’ sex, household economic status, and children’s age. The implementation of future educational campaigns is essential to enhance parental understanding of medications for children, address their concerns, and promote rational and safe medication administration practices. Future research should adopt a multidimensional analysis to enhance the characterization of parental attitudes toward the administration of medicines to children.

## Figures and Tables

**Table 1:** Parent sociodemographic characteristics.

Variable	Category	N	%
Sex	Female	837	71.2
	Male	338	28.8
Age (years)	<25	151	12.9
	25–34	300	25.5
	35–44	402	34.2
	45–55	227	19.3
	>55	95	8.1
Education level	High school or lower	305	26.0
	Diploma	304	25.9
	Bachelor’s degree	477	40.6
	Postgraduate	89	7.6
Occupation	Not working	414	35.2
	Student	139	11.8
	Working	509	43.3
	Retired	113	9.6
Marital status	Married	981	83.5
	Widowed	80	6.8
	Divorced	114	9.7
Monthly income (JOD)	<500	459	39.1
	500–1000	507	43.1
	1001–1500	172	14.6
	>1500	37	3.1

JOD: Jordanian dinar; 1 JOD equals 1.41 USD.

**Table 2:** Child and health-related characteristics.

Variable	Category	N	%
Age of child(ren) *			
Child’s age 0–2 years	Yes	329	28.0
Child’s age 3–5 years	Yes	330	28.1
Child’s age 6–8 years	Yes	327	27.8
Child’s age 9–12 years	Yes	321	27.3
Child’s age 13–18 years	Yes	304	25.9
Number of children	1	232	19.7
	2	326	27.7
	3	312	26.6
	4 or more	305	26.0
Comorbidities	None	928	79.0
	One or more	247	21.0
Frequency of acute illnesses in children (e.g., fever, cold, infections)	Sometimes (1–3 times/year)	638	54.3
	Frequently (≥4 times/year)	176	15.0
	Rarely	361	30.7
Child hospitalized in the past 12 months	No	880	74.9
	Yes	295	25.1
Do you usually consult a physician before giving medication to your child?	Never	155	13.2
	Rarely	163	13.9
	Sometimes	268	22.8
	Often	269	22.9
	Always	320	27.2
Have you ever given your child OTC medication without consulting a physician?	No	771	65.6
	Yes	404	34.4
Main source of information about pediatric medications	Friends or family	59	5.0
	Internet	87	7.4
	Pharmacist	446	38.0
	Physician	566	48.2
	Media	17	1.4
Do you or your partner work in healthcare?	No	889	75.7
	Yes	286	24.3

* This question allowed multiple selections.

**Table 3:** Multiple linear regression analysis findings.

Coefficients ^a^								
Model		Unstandardized Coefficients		Standardized Coefficients	t	Sig.	95% Confidence Intervals for B	
		B	Std. Error	Beta			Lower Bound	Upper Bound
1	(Constant)	49.563	1.500		33.042	<0.001	46.620	52.506
	Sex	−2.060	0.828	−0.075	−2.489	0.013	−3.684	−0.436
	Age	−0.758	0.404	−0.068	−1.877	0.061	−1.550	0.034
	Education level	0.813	0.473	0.061	1.718	0.086	−0.115	1.742
	Occupation	0.457	0.418	0.038	1.093	0.275	−0.364	1.278
	Marital status	−1.104	0.588	−0.055	−1.877	0.061	−2.258	0.050
	Income	1.521	0.550	0.097	2.763	0.006	0.441	2.600
	Age of children							
	0–2 years	1.852	1.022	0.067	1.812	0.070	−0.153	3.857
	3–5 years	1.821	0.916	0.066	1.987	0.047	0.023	3.618
	6–8 years	0.175	0.888	0.006	0.197	0.844	−1.567	1.917
	9–12 years	0.230	0.904	0.008	0.255	0.799	−1.544	2.004
	13–15 years	0.234	1.072	0.008	0.218	0.828	−1.869	2.336
	Number of children	0.123	0.419	0.011	0.294	0.769	−0.700	0.946
	Comorbidities	−1.539	0.906	−0.050	−1.698	0.090	−3.317	0.239

^a^. Dependent Variable: total attitude score.

## Data Availability

The data presented in this study are available upon request from the corresponding author. The data are not publicly available due to privacy restrictions.

## Supplementary Materials

The following supporting information can be downloaded at: https://www.mdpi.com/article/10.3390/healthcare14152288/s1, File S1: Parental Attitudes Toward Medicine Use in Children Questionnaire; Table S1: Rotated Component Matrix.
