# Supplementary material for: Parental Attitudes Toward the Administration of Medicines to Children: A Cross-Sectional Study in Jordan
Source: Healthcare (Basel). 2026 Jul 28;14(15):2288. doi: 10.3390/healthcare14152288 (PMC13465365; doi:10.3390/healthcare14152288)
Supplement: Supplementary file 1 [file healthcare-14-02288-s001.zip › healthcare-4437701-supplementary.pdf]

Supplementary Material

File S1. Parental Attitudes Toward Medicine Use in Children Questionnaire

Purpose

This questionnaire aims to explore parents' attitudes toward the use of medicines in their children, as well as background information that may influence these attitudes. You are requested to respond to attitude items regarding your children's medication use without specifying a particular referent child, based on your overall experience. All responses are confidential and will be used for research purposes only.

Section A: Sociodemographic and Background Information

Please tick (✓) or fill in the option that applies to you.

Parent Information

1. **Parent's gender:**
   ☐ Male ☐ Female ☐ Other
2. **Age:**
   ☐ Under 25 ☐ 25–34 ☐ 35–44 ☐ 45–54 ☐ 55 or above
3. **Highest educational level completed:**
   ☐ Primary school ☐ Secondary school ☐ Bachelor's degree ☐ Master's or higher
4. **Occupation:**
   ☐ Employed ☐ Unemployed ☐ Student ☐ Retired
5. **Marital status:**
   ☐ Single ☐ Married ☐ Divorced ☐ Widowed
6. **Family monthly income (approximate):**
   ☐ Less than 500 JD ☐ 501-1000 JD ☐ 1001-1500 JD ☐ 1501 JD and above

---

Child Information

7. **Children's age:**
   ☐ 0–2 years ☐ 3–5 years ☐ 6–8 years ☐ 9–12 years ☐ 13–18 years
8. **Number of children you have:**
   ☐ 1 ☐ 2 ☐ 3 ☐ 4 or more

Health and Disease History

11. **Does your child(ren) currently have any chronic illness (e.g., asthma, diabetes, allergies)?**
    ☐ Yes ☐ No

12. **How often does your child(ren) experience acute illnesses (e.g., fever, colds, infections)?**
    ☐ Rarely ☐ Occasionally (1–3 times/year) ☐ Often (4+ times/year)

13. **Has your child(ren) been hospitalized in the past 12 months?**
    ☐ Yes ☐ No

14. **Do you usually consult a physician before giving medicines to your child(ren)?**
    ☐ Always ☐ Often ☐ Sometimes ☐ Rarely ☐ Never

15. **Have you ever given your child(ren) over-the-counter (OTC) medicines without consulting a doctor?**
    ☐ Yes ☐ No

16. **Main source of information about children's medicines:**
    ☐ Doctor ☐ Pharmacist ☐ Internet ☐ Friends/family ☐ Media
    ☐ Other: ____________

17. **Do you or another parent work in a healthcare profession?**
    ☐ Yes ☐ No

Section B: Parental Attitudes Toward Medicine Use in Children

Instructions:
Please indicate how strongly you agree or disagree with each statement.

Scale:

1	2	3	4	5	No opinion
Strongly Disagree	Disagree	Neutral	Agree	Strongly Agree	

Component 1: General Attitude Toward Medicines

No.	Statement	1	2	3	4	5	No opinion
1	"Fever, a natural means of defense of the child's body, should not be lowered artificially with medicines. "						
2	"The child needs to learn how to bear pain. "						
3	"I try to take care of my child's ailments by some other means than using medicines. "						
4	"I try to avoid giving medicines to my child. "						
5	"I usually give less analgesics to the child than recommended in the instructions. "						
6	"Medicines can disturb the body's own capability to heal illnesses. "						
7	"Medicines are unnatural to the human body. "						

Component 2: Attitude Toward Prescription Medicines

No.	Statement	1	2	3	4	5	No opinion
8	"Prescription medicines are effective. "						
9	Prescription medicines are safe. "						
10	"Medicines that a doctor has prescribed for the child are necessary. "						
11	"Medicines are necessary in treating illnesses. "						

Component 3: Attitude Toward Over-the-Counter (OTC) Medicines

No.	Statement	1	2	3	4	5	No opinion
12	"OTC medicines are effective. "						
13	"I take care of my child's minor ailments by using OTC medicines. "						
14	"Over-the-counter (OTC) medicines are safe. "						

Component 4: Attitude Toward the Risks of Medicines

No.	Statement	1	2	3	4	5	No opinion
15	"Interactions between medicines worry me. "						
16	"Side effects of children's medicines worry me. "						

Component 5: Attitude Toward Long-Term Use of Painkillers

No.	Statement	1	2	3	4	5	No opinion
17	"Long-term use of analgesics reduces the pain threshold. "						
18	"The more you need to use analgesics, the less effective they are against pain. "						

Table S1. Rotated Component Matrix

Parental Attitude Item	Factor 1	Factor 2	Factor 3	h^2
Factor 1: Beliefs and Concerns Regarding Medications				
"Medications prescribed by a physician for a child are necessary"	0.798	—	—	0.681
"Medications are necessary for treating diseases"	0.795	—	—	0.681
"Prescribed medications are safe"	0.767	—	—	0.625
"Prescribed medications are effective"	0.761	—	—	0.635
"Drug–drug interactions concern me" [R]	0.719	—	—	0.655
"The side effects of pediatric medications concern me" [R]	0.717	—	—	0.636
"Long-term use of pain relievers reduces the body's ability to tolerate pain" [R]	0.678	0.316	—	0.604
"The more pain relievers are used, the less effective they become in relieving pain" [R]	0.665	0.316	—	0.598
Factor 2: Medication Avoidance and Naturalistic Beliefs				
"I try to treat my child's illnesses using methods other than medications" [R]	—	0.730	—	0.622
"A child should learn to tolerate pain" [R]	—	0.714	—	0.546
"I try to avoid giving medications to my child as much as possible" [R]	0.348	0.684	—	0.590
"Medications are unnatural to the human body" [R]	—	0.642	—	0.542
"Fever is a natural defense mechanism of the child's body and should not be artificially reduced with medications" [R]	—	0.630	—	0.506
"Medications can negatively affect the body's natural ability to heal" [R]	0.323	0.622	—	0.538
"I usually give my child lower doses of pain relievers than those stated in the instructions" [R]	—	0.610	0.437	0.563
Factor 3: Confidence in OTC Medications				
"Over-the-counter medications are safe"	—	—	0.818	0.734
"Over-the-counter medications are effective"	—	—	0.746	0.655
"I manage my child's minor illnesses using over-the-counter medications"	0.314	—	0.681	0.633
Eigenvalue	5.001	3.668	2.374	—
% of Variance Explained	27.78%	20.38%	13.19%	—
Cumulative % of Variance	27.78%	48.16%	61.35%	—

R: Reversely-coded item before estimating the total attitude score
